# Prevalence and Severity of Pruritus in Patients on Hemodialysis: A Cross-Sectional Study

**DOI:** 10.1177/20543581251380541

**Published:** 2025-09-28

**Authors:** Hannah G. McMaster, Rachel M. Holden, Melissa Scott, Eduard Iliescu

**Affiliations:** 1 Department of Medicine, Queen’s University, Kingston, ON, Canada; 2 Department of Nephrology, Kingston Health Sciences Centre, ON, Canada

**Keywords:** hemodialysis, pruritus, itching, symptoms, quality of life

## Abstract

**Background:**

Chronic kidney disease-associated pruritus (CKD-aP) is a distressing symptom associated with dialysis that negatively affects quality of life. Chronic kidney disease-associated pruritus is under-recognized due to a lack of clinical attention and symptom screening.

**Objective:**

Assess the prevalence and severity of CKD-aP in a regional hemodialysis program.

**Design:**

Cross-sectional study.

**Setting and Patients:**

All outpatients receiving in-center hemodialysis at the Kingston Health Sciences Centre.

**Measurements:**

Patients were asked to complete the Worst Itching Intensity Numerical Scale (WI-NRS), with moderate-to-severe pruritus classified as a score greater than 4, and the Self-Assessed Disease Severity (SADS) scale. Demographic, laboratory, and prescription data were extracted from patient medical records and patients were asked to self-report over-the-counter pruritus medications.

**Methods:**

Comparative differences in demographics and laboratory values at the time of determining the WI-NRS and SADS were analyzed using a Fisher’s exact test with Bonferroni correction for categorical variables and the Mann-Whitney *U* test for continuous variables. Correlations between select variables and the WI-NRS score were assessed using linear regression analyses. Adjusted associations with moderate-to-severe CKD-aP were examined using odds ratios with corresponding 95% confidence intervals.

**Results:**

A total of 307 patients completed the WI-NRS and 302 completed the SADS. Fifty-seven percent of patients reported some degree of CKD-aP, 31% of patients had moderate-to-severe CKD-aP, and 9% reported interference with quality of life (patients in SADS group C). Patients with moderate-to-severe CKD-aP and those significantly affected by CKD-aP (patients in SADS group C) were more likely to use over-the-counter treatments than patients with mild or no CKD-aP (*P* < .0001) and patients in SADS group A (*P* < .0001), respectively. Of patients with moderate-to-severe CKD-aP and whose CKD-aP significantly affected their quality of life (patients in SADS group C), 42% and 11.11%, respectively, did not use any form of treatments. Patients with moderate-to-severe CKD-aP had significantly higher parathyroid hormone (PTH; 0.02) and phosphate (*P* = .01). A higher body mass index (BMI) was associated with a greater WI-NRS score (*R*^2^ = 0.030, *P* = .003). Of patients with moderate-to-severe CKD-aP, 24% reported significant debilitation (patients in SADS group C). Finally, adjusted associations were found between moderate-to-severe CKD-aP and the following variables: BMI (OR = 1.05, 95% CI = 1.01-1.09, *P* = .02); serum phosphate (OR = 2.12, 95% CI = 1.15-4.00, *P* = .02); being a current smoker (OR = 0.46, 95% CI = 0.20-0.95, *P* = .04); and a serum phosphate greater than or equal to 1.8 mmol/L (OR = 2.33, 95% CI = 1.29-4.26, *P* = .01).

**Limitations:**

There were some missing data points in patient records and patients’ reports of over-the-counter medications. We could not assess whether patients actually had CKD-aP, or pruritus due to other causes. Treatment adherence could not be measured as well as whether treatments were specifically prescribed for CKD-aP. Moreover, our electronic medical record system could not capture prescribed topicals or pruritus-related medical conditions. Finally, this study did not assess physician’s awareness of CKD-aP.

**Conclusions:**

A substantial proportion of patients with moderate-to-severe CKD-aP reported a significant impact on quality of life. Elevated PTH, phosphate, and BMI were associated with CKD-aP. In addition, almost half of patients with moderate-to-severe CKD-aP did not use any treatments. There exist gaps and opportunities for care for patients with CKD-aP. Increased clinical attention to CKD-aP could identify those who may benefit from care interventions that improve quality of life.

## Abrégé

**Contexte:**

Le prurit associé à l’insuffisance rénale chronique (Pa-IRC) est un symptôme éprouvant lié à la dialyse qui affecte négativement la qualité de vie. Le Pa-IRC demeure méconnu, faute de dépistage et de suivi clinique adaptés.

**Objectif:**

Examiner la prévalence et la gravité du Pa-IRC dans un programme régional d’hémodialyse.

**Conception:**

Étude transversale

**Cadre et sujets de l’étude:**

Tous les patients ambulatoires recevant l’hémodialyse au Kingston Health Sciences Centre.

**Mesures:**

On a demandé aux patients d’évaluer leurs démangeaisons sur l’échelle WI-NRS (Worst Itching Intensity Numerical Scale) — où un score supérieur à 4 indique un prurit modéré à sévère — et de compléter l’évaluation SADS (Self-assessed disease severity) qui mesure la gravité perçue de la maladie. Les données démographiques, les résultats de laboratoire et les prescriptions des patients ont été extraits des dossiers médicaux. Les patients ont également été invités à auto-déclarer les médicaments en vente libre pris pour soulager le prurit.

**Méthodologie:**

Les différences entre les caractéristiques démographiques et les paramètres biologiques, recueillis au moment des évaluations WI-NRS et SADS, ont été analysées par le test exact de Fisher (avec correction de Bonferroni) pour les variables catégorielles, et le test de Mann-Whitney U pour les variables continues. Des analyses de régression linéaire ont servi à déterminer les corrélations entre les variables sélectionnées et le score WI-NRS. Les associations ajustées avec le Pa-IRC modéré à sévère ont été examinées par rapports de cotes (RC) avec intervalles de confiance à 95 % correspondants.

**Résultats:**

En tout, 307 patients ont complété l’évaluation WI-NRS et 302 ont complété l’évaluation SADS. De ceux-ci, 57 % ont déclaré souffrir d’un certain degré de Pa-IRC, 31 % ont signalé un Pa-IRC modéré à sévère et 9 % ont déclaré que le Pa-IRC affectait leur qualité de vie (patients du groupe C dans l’évaluation SACS). Les patients souffrant de Pa-IRC modéré à sévère, ainsi que ceux qui se disaient significativement affectés par le Pa-IRC (groupe C dans SADS), étaient respectivement plus susceptibles d’avoir recours aux traitements en vente libre que les patients ayant signalé l’absence de Pa-IRC ou une légère affection (p<0,0001) et que les patients du groupe A de l’évaluation SADS (p<0,0001). Par ailleurs, 42 % des patients souffrant de Pa-IRC modéré à sévère et 11,11 % des patients dont le Pa-IRC affecte significativement la qualité de vie (groupe C dans SADS) ne prenaient aucune forme de traitement. Les patients souffrant de Pa-IRC modéré à sévère présentaient des taux plus élevés de PTH (0,02) et de phosphate (p=0,01). Un IMC plus élevé a été associé à un score WI-NRS plus élevé (R^2^=0,030; p=0,003). Parmi les patients souffrant de Pa-IRC modéré à sévère, 24 % ont déclaré une limitation fonctionnelle importante (groupe C dans SADS). Enfin, des associations ajustées ont été observées entre le Pa-IRC modéré à sévère et les variables suivantes: l’IMC (RC=1,05; IC95: 1,01-1,09; p=0,02); le taux de phosphate sérique (RC=2,12; IC95: 1,15-4,00; p=0,02); le tabagisme (RC=0,46 IC95: 0,20=0,95; p=0,04); un taux de phosphate sérique supérieur ou égal à 1,8 mmol/l (RC=2,33; IC95: 1,29-4,26; p=0,01).

**Limites:**

Certaines données manquaient dans les dossiers médicaux, ainsi que dans les auto-déclarations concernant les médicaments en vente libre. Nous n’avons pas pu évaluer si les patients présentaient réellement un Pa-IRC ou un prurit dû à d’autres causes. Il n’a pas été possible de mesurer l’observance du traitement ni de déterminer si les médicaments étaient prescrits spécifiquement pour soulager le Pa-IRC. Notre système de dossiers électroniques ne permettait pas de relever les traitements topiques prescrits ou les maladies liées au prurit. Enfin, l’étude n’a pas évalué la sensibilisation des médecins au Pa-IRC.

**Conclusion:**

Une importante proportion de patients souffrant de Pa-IRC modéré à sévère a déclaré que ce symptôme affectait significativement la qualité de vie. Le Pa-IRC a été associé à un IMC et des taux de PTH et de phosphate élevés. Aussi, près de la moitié des patients souffrant de Pa-IRC modéré à sévère ne prenaient aucun traitement. Il subsiste des lacunes dans la prise en charge du Pa-IRC, mais il existe aussi des opportunités d’amélioration. Porter une attention clinique accrue à ce symptôme aiderait à cibler les patients susceptibles de bénéficier d’interventions visant à améliorer la qualité de vie.

## Introduction

Chronic kidney disease-associated pruritus (CKD-aP) is a debilitating symptom affecting many patients with CKD^1–4^ and patients receiving dialysis.^1,4–7^ The Dialysis Outcomes and Practice Patterns Study (DOPPS) found that 43% of patients in Canada and 42% globally experience moderate-to-severe pruritus.^5^

CKD-aP is a distressing condition where itching often provides little relief.^1–4^ It is associated with poor sleep,^8^ depression, mental health problems,^9,10^ as well as an overall reduction in quality of life (QOL).^11^ Studies have shown a link between CKD-aP and increased mortality.^5,12^ CKD-aP also interferes with dialysis treatment itself, having been associated with increased dialysis treatment recovery time and an increased likelihood of withdrawing from dialysis.^1,7^

Due to significant effects of CKD-aP on QOL, effective symptom management is important. However, CKD-aP is underrecognized and undertreated by both patients and health care professionals. Rayner et al reported that 17% of patients on hemodialysis consistently experiencing itch symptoms did not report them to health care professionals.^4^ Lack of symptom reporting may occur due to insufficient patient knowledge regarding the relationship between CKD and pruritus, the lack of prompts for discussion during clinical assessment, as well as attitudes regarding the significance of itch as a symptom.^13,14^ There are also discrepancies between physician and patient reported rates of CKD-aP.^4,15,16^ In a study of 35 452 patients receiving hemodialysis, 69% of medical facilities’ directors underestimated the prevalence of pruritus.^4^ Delayed detection of CKD-aP can also occur when visible skin lesions are absent.^4,13^ Guidelines from Kidney Disease Improving Global Outcomes (KDIGO) recommend performing routine symptom screening to improve QOL^17^; however, there exists a lack of clinical attention to CKD-aP.

Treatments for CKD-aP may include skin hydration through various topical agents, antihistamines, and other prescription medications.^18–23^ Several studies have shown anticonvulsants such as Pregabalin and Gabapentin to be effective treatments for CKD-aP.^3,18,21,24,25^ However, as these drugs are cleared by the kidneys and their half-life is increased with hemodialysis, they have risks of neurological adverse effects including dizziness, falls, and drowsiness.^21^ Antihistamines present an option as first- or second-line pruritus treatments^18^; however, several studies have shown them to be not effective in reducing itch, with possible safety concerns of sedative effects.^21,26,27^ Difelikefalin, a peripherally acting kappa-opioid receptor agonist, was recently approved as a first line treatment for CKD-aP due to its success in reducing pruritus in patients on hemodialysis in recent clinical trials.^28–30^ However, it is limited by its high cost.^31^ Finally, increasing literature has suggested the use of cannabinoids for CKD-aP, although further research is warranted as the majority of these studies were preliminary.^32^ In the study by Rayner et al^4^ of more than 30 000 patients on hemodialysis across 17 countries, the most common first line treatment for CKD-aP was antihistamines, with Gabapentin and other prescribed medications more common as second- or third-line treatments, and few health care professionals prescribed opioids or cannabinoids.

While there do exist a variety of treatments for CKD-aP, they are limited by a lack of understanding of their mechanisms of action, are ineffective in many patients, and there exist few recommendation treatment guidelines.^18,21^ Moreover, the etiology and pathogenesis of CKD-aP itself is not well understood. CKD-aP is multifactorial and has been linked to abnormalities in phosphate, calcium and PTH, systemic inflammation, co-morbidities including diabetes, and accumulation of uremic toxins.^33–38^

Due to the current lack of recognition, diagnosis, and treatment of CKD-aP, the over-arching objective of this study was to examine the prevalence and severity of CKD-aP in a regional hemodialysis program. Our specific objectives were (1) to administer 2 CKD-aP-associated itch scales to all patients receiving hemodialysis, (2) to identify factors associated with moderate-to-severe CKD-aP, and (3) to evaluate the use of available therapies by patients within our program. Ultimately, we wished to identify whether available tools could identify patients who might benefit from a care intervention designed to improve QOL.

## Methods

All outpatients receiving in-center hemodialysis at the Kingston Health Sciences Centre who received a monthly blood draw in July 2024 were asked to complete 2 pruritus intensity scales (see supplement). The Worst Itching Intensity Numerical Scale (WI-NRS) is a validated 10-point scale with a score of more than 4 points corresponding to moderate-to-severe pruritus according to previous literature.^29,39,40^ In alignment with previous studies, moderate-to-severe CKD-aP was defined as a WI-NRS score ≥ 5, and mild or no CKD-aP as a WI-NRS score < 5. The Self-Assessed Disease Severity (SADS) scale captures the multidimensional effects of CKD-aP on QOL and sleep.^6^ Patients categorize themselves into A, B, and C which reflects an increasing severity and impact of CKP-aP on QOL and sleep.^6^ The SADS scale has been validated against other measures of sleep, QOL, and disease severity.^6^

A cross-sectional study design was used and the following data were extracted from patient medical records: demographic data of age, sex, body mass index (BMI), type I diabetes, type II diabetes, and current smoking patterns; laboratory data of parathyroid hormone (PTH), alkaline phosphatase (ALP), alanine transaminase (ALT), calcium, ferritin, hemoglobin, phosphate, transferrin saturation (TSAT), urea reduction ratio (URR), and vitamin D; and prescription data of common pruritus medications available in our electronic medical record system including Gabapentin, Pregabalin, Hydroxyzine, and Cetirizine. Patients were asked to self-report over-the-counter (OTC) pruritus medications including creams and antihistamines (see supplement). As this was a practice audit, waived consent was obtained to collect all data from medical records as per the Queen’s University Health Sciences and Affiliated Teaching Hospitals Research Ethics Board (REB [REB #6040038]).

All statistical analysis was performed in GraphPad Prism (V.10.1.0, GraphPad Software, La Jolla, CA) and Microsoft Excel (V.16.93.1, Microsoft Corporation, Redmond, WA). Statistical significance was considered at p < 0.05. Normal distribution was checked using the Shapiro-Wilk test. Characteristic differences in age, BMI and all laboratory variables between categories of pruritus severity, WI-NRS ≥ 5 and < 5, were compared using non-parametric *t*-tests (Mann-Whitney *U* tests). Differences in sex, type I diabetes, type II diabetes, current smoking patterns, all OTC and prescription medications, and SADS classifications between categories of pruritus severity were compared using Fisher’s exact tests with a Bonferroni correction. Linear regressions were performed between WI-NRS score (dependent variable) and all laboratory data, age, and BMI (independent variables). Finally, adjusted odds ratios (OR) and 95% confidence intervals (CI) for moderate-to-severe CKD-aP (WI-NRS ≥ 5) were calculated for variables of age, sex, BMI, type I diabetes, type II diabetes, smoking patterns and all laboratory data.

Primary outcomes were prevalence of CKD-aP overall; prevalence of moderate-to-severe CKD-aP; prevalence of significant impact of CKD-aP on QOL overall; and prevalence of significant impact of CKD-aP on QOL within patients with moderate-to-severe CKD-aP.

## Results

### Prevalence and Severity of CKD-aP

Of 372 outpatients who received a blood draw at our in-center hemodialysis unit in July 2024, 307 filled out the WI-NRS and 302 filled out the SADS. A total of 174 patients (57%) reported at least some degree of pruritus (WI-NRS > 0) and 95 patients (31%) had moderate-to-severe CKD-aP (WI-NRS ≥ 5). Two hundred patients (66%) classified themselves in SADS group A, 75 (25%) group B and 27 (9%) group C (most severe impact of CKD-aP on QOL) (Table 1). The median WI-NRS score in patients with mild or no CKD-aP was 0 (interquartile range [IQR], 0-2) and in patients with moderate-to-severe CKD-aP was 7 (IQR, 5-8).

**Table 1.** Demographic and Laboratory Variables in Mild or No CKD-aP Versus Moderate-to-Severe CKD-aP.

	Overall (n = 307)	WI_NRS < 5 (n = 212)	WI_NRS ≥ 5 (n = 95)	Test for comparison between WINRS < 5 and WINRS ≥ 5
**Demographics**				
Age (years), median (IQR)	69 (61-78)	70 (61-78)	68 (59-76)	*P* = .16
Male, n (%)	173/307 (56%)	125/212 (59%)	48/95 (51%)	*P* = .17
BMI (kg/m^2^), median (IQR)	26.95 (23.20-32.20)	26.40 (22.95-31.30)	27.80 (23.75-34.10)	*P* = .08
T1 diabetes, n (%)	28/298 (9)	15/205 (7)	13/93 (14)	*P* = .09
T2 diabetes, n (%)	137/299 (46)	37/206 (18)	37/93 (40)	*P* = .17
Current smoker, n (%)	59/284 (21)	45/196 (23)	14/88 (16)	*P* = .21
**Laboratory data**				
PTH (pmol/L), median (IQR)	41.20 (25.60-65.65)	37.50 (23.00-63.55)	46.60 (27.95-71.80)	*P* = **.02**
ALP (U/L), median (IQR)	113.0 (90.00-160.3)	110.0 (91.00-154.5)	123.0 (85.00-168.0)	*P* = .20
ALT (U/L), median (IQR)	13 (10-19)	14 (10-19)	13 (10-19)	*P* = .44
Calcium (mmol/L), median (IQR)	2.34 (2.21-2.45)	2.35 (2.22-2.45)	2.34 (2.21-2.46)	*P* = .92
Ferritin (ng/mL), median (IQR)	360.0 (180.8-615.8)	357.0 (186.3-609.3)	363.5 (169.8-624.0)	*P* = .95
Hemoglobin (g/L), median (IQR)	112.0 (106.0-120.0)	112.0 (106.0-161.0)	112.5 (105.0-145.0)	*P* = .92
Phosphate (mmol/L), median (IQR)	1.64 (1.36-1.95)	1.60 (1.33-1.85)	1.70 (1.47-2.10)	*P* = **.01**
TSAT, median (IQR)	24 (19-31)	24 (19-31)	24 (19-30)	*P* > .99
URR, median (IQR)	0.75 (0.70-0.79)	0.75 (0.70-0.79)	0.74 (0.70-0.80)	*P* = .98
Vitamin D (nmol/L), median (IQR)	97.80 (72.70-120.0)	98.00 (75.50-123.3)	95.35 (65.00-115.6)	*P* = .30
**SADS Group**				
A, n (%)	200/302 (66)	173/209 (83)	27/93 (29)	A vs B **(***P <* **.0001)**
B, n (%)	75/302 (25)	31/209 (15)	44/93 (47)	A vs C (*P <* **.0001**)
C, n (%)	27/302 (9)	5/209 (2)	22/93 (24)	B vs C (*P* = .04)

Abbreviations: WI_NRS = Worst Itching Intensity Numerical Scale; IQR = interquartile range; BMI = body mass index; T1 = type I; T2 = type II; PTH = parathyroid hormone; ALP = alkaline phosphatase; ALT = alanine transaminase; TSAT = transferrin saturation; URR = urea reduction ratio; kg = kilogram; m = meter; pmol = picomole; L = liter; U = unit; mmol = millimole; ng = nanogram; mL = millimeter; g = gram; nmol = nanomole.

**Figure 1.**
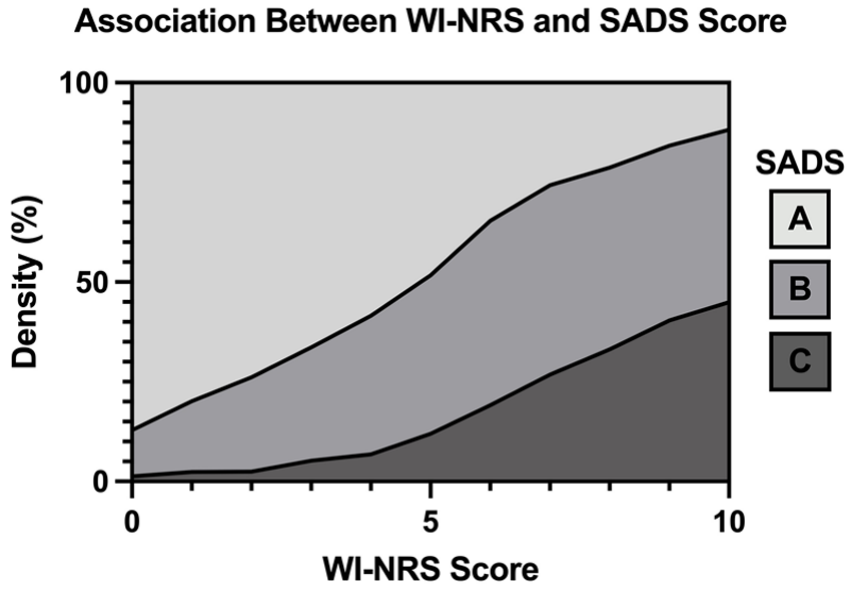
Association between WI-NRS and SADS score. As WI-NRS score increases, the impact of CKD-aP on QOL also increases (higher prevalence of SADS group B and C). Abbreviations: WI-NRS = Worst Itching Intensity Numerical Scale; SADS = Self-Assessed Disease Severity.

Of the 95 patients with moderate-to-severe CKD-aP, 27 patients classified themselves in SADS group A (29%), 44 in group B (47%) and 22 in group C (24%). Patients with moderate-to-severe CKD-aP were more likely to classify themselves into group B (47% vs 15%, *P* < .0001) and C (24% vs 2%, *P* < .0001) than group A compared with those with mild or no CKD-aP (Table 1). The mean WI-NRS score in patients who reported a significant effect of CKD-aP on QOL (SADS group C) was significantly higher than patients in SADS group A (7.11 ± 3.15 vs 1.44 ± 2.16, *P* < .0001), and the mean WI-NRS score in patients in SADS group A was significantly smaller than patients in SADS group B (1.44 ± 2.16 vs 4.87 ± 2.98, *P* < .0001). Figure 1 depicts the association between WI-NRS and SADS scores.^41^

### Therapies for Patients with Moderate-to-Severe CKD-aP

**Figure 2.**
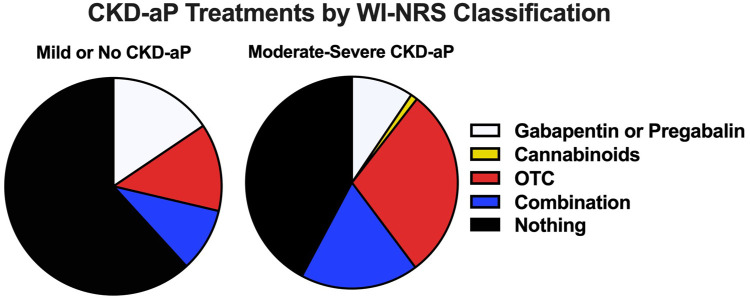
Treatments received by patients with mild or no CKD-aP versus moderate-to-severe CKD-aP. Abbreviations: CKD-aP = chronic kidney disease-associated pruritus; WI-NRS = Worst Itching Intensity Numerical Scale; OTC = over the counter.

Patients reporting moderate-to-severe CKD-aP were more likely to be using OTC medications compared with those with mild or no CKD-aP (42% vs 12%, *P* < .0001), and specifically were more likely to use antihistamines (*P* < .0001) or an antihistamine and cream combination (*P* = .02) compared with those with mild or no CKD-aP who were more likely to be using only cream. Of those with moderate-to-severe CKD-aP, 29% were using OTC medications, 1% were using cannabinoids, 9% were taking prescribed Gabapentin or Pregabalin, and 18% were using a combination of these therapies (of these patients 1 was prescribed antihistamines in addition to other treatments). Forty-two percent were using no treatments (Figure 2).

**Figure 3.**
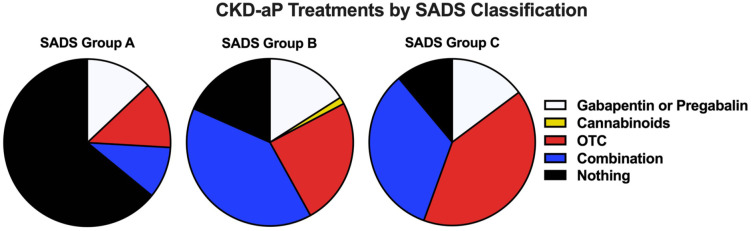
Treatments received by patients according to SADS scale classification. Abbreviations: CKD-aP = chronic kidney disease-associated pruritus; SADS = Self-Assessed Disease Severity; OTC = over the counter.

Patients with a significant impact of CKD-aP on QOL (SADS group C) were likewise more likely to use OTC medications compared with group A (67% vs 12%, *P* < .0001) and B (67% vs 33%, *P* = .003) and those in group B were more likely to use OTC medications compared with group A (33% vs 12%, *P* = .0002). In addition, patients with a severe QOL-impact (SADS group C) were more likely to use Pregabalin compared with those in SADS group B (41% vs 17%, *P* = .0002) and SADS group A (41% vs 13%, *P* < .0001). Within SADS group C, 41% were using OTC medications, 14% were taking prescribed Gabapentin or Pregabalin, 33% were using a combination of these therapies, 11% were using no treatments and no patients were prescribed antihistamines or were using cannabinoids (Figure 3).

### Associations with CKD-aP

**Figure 4.**
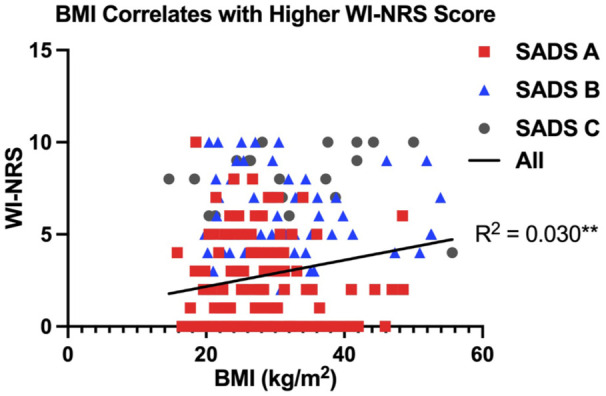
WI-NRS score is associated with BMI. BMI associates with WI-NRS score in all patients (*R*^2^ = 0.030, *P* = .0034). Linear regression test (*P < .05, **P < .01). Abbreviations: BMI = body mass index; WINRS = Worst Itching Intensity Numerical Scale; SADS = Self-Assessed Disease Severity.

**Figure 5.**
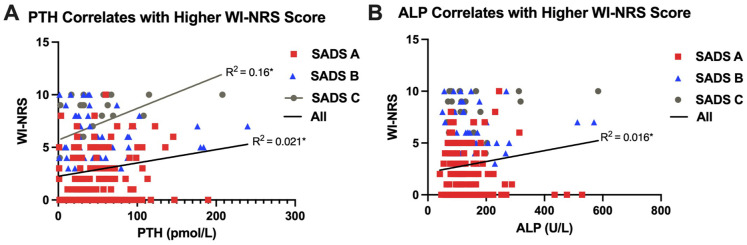
WI-NRS score is associated with PTH and ALP. (A) PTH associates with WI-NRS score in all patients (*R*^2^ = 0.021, *P* = .016) and in the SADS group C (*R*^2^ = 0.16, *P* = .041). (B) ALP associates with WI-NRS score in all patients (*R*^2^ = 0.016, *P* = .034). Linear regression tests (*P < .05). Abbreviations: PTH = parathyroid hormone; WI-NRS = Worst Itching Intensity Numerical Scale; SADS = Self-Assessed Disease Severity; ALP = alkaline phosphatase.

**Figure 6.**
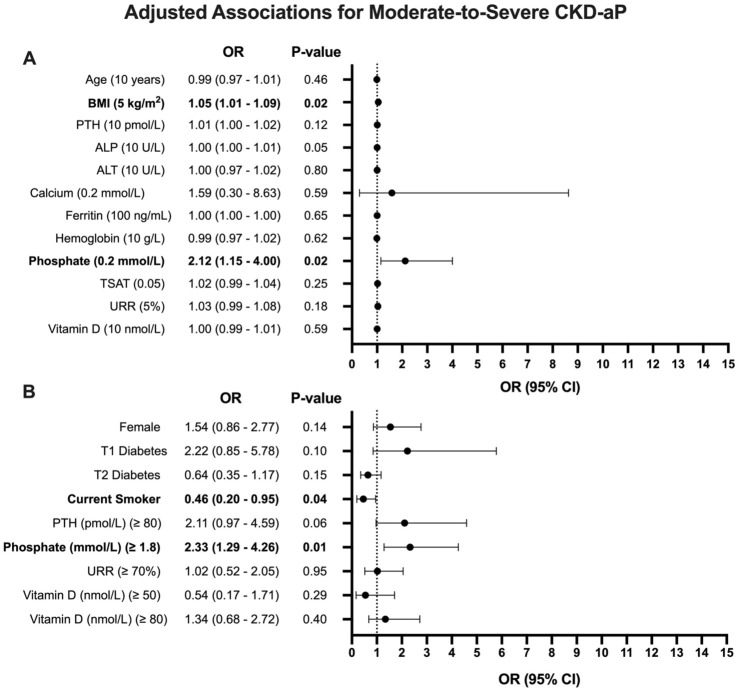
Adjusted associations for moderate-to-severe CKD-aP. OR presented with 95% CI. (A) OR for continuous variables calculated using logistic regression with incremental increases appropriate for each variable, as indicated in the figure. (B) OR for categorical variables calculated using logistic regression for dichotomous variables with predetermined thresholds, as indicated in the figure. Abbreviations: CKD-aP = chronic kidney disease-associated pruritus; OR = odds ratio; CI = confidence interval; BMI = body mass index; T1 = type I; T2 = type II; PTH = parathyroid hormone; ALP = alkaline phosphatase; ALT = alanine transaminase; TSAT = transferrin saturation; URR = urea reduction ratio; kg = kilogram; m = meter; pmol = picomole; L = liter; U = unit; mmol = millimole; ng = nanogram; mL = millimeter; g = gram; nmol = nanomole.

A higher WI-NRS score was associated with having an increased BMI (*R*^2^ = 0.030, *P* = .003) (Figure 4). Patients with moderate-to-severe CKD-aP also had a higher PTH (*P* = .02) and phosphate (*P* = .01) compared with those with mild or no CKD-aP (Table 1). In addition, there was a significant association between WI-NRS score and PTH (*R*^2^ = 0.021, *P* = .02) and ALP (*R*^2^ = 0.016, *P* = .03) (Figure 5). Adjusted associations for CKD-aP included a higher BMI (OR = 1.05, 95% CI = 1.01-1.09, *P* = .02), a higher serum phosphate (OR = 2.12, 95% CI = 1.15-4.00, *P* = .02), being a current smoker (OR = 0.46, 95% CI = 0.20-0.95, *P* = .04), and a serum phosphate greater than or equal to 1.8 mmol/L (OR = 2.33, 95% CI = 1.29-4.26, *P* = .01) (Figure 6).

## Discussion

Overall, over half of patients in our study had some degree of pruritus, more than a quarter had moderate-to-severe pruritus and just under 10% reported that pruritus significantly affected their QOL. Moreover, just under half of patients with moderate-to-severe CKD-aP and more than 10% of patients whose QOL was severely affected were not using any form of treatment.

The prevalence of pruritus in this study was 57%. This is supported by previous literature that found the prevalence of pruritus in patients on hemodialysis to be 50.1% in Austrian patients^41^ and 55% in a meta-analysis of 42 international studies.^37^ However, as highlighted by the DOPPS study, the prevalence of pruritus in patients on hemodialysis can vary between 26% and 48% in different countries.^5^ In our study, 31% of patients reported moderate-to-severe CKD-aP. The DOPPS study^5^ however found that 43% of patients on hemodialysis in Canada and 42% overall were moderately to extremely bothered by itch. This may be due to the fact that the WI-NRS scale assesses itch in the past 24 hours and the DOPSS study assessed a larger time frame of the past 4 weeks.^5^ Although, another study by Engler et al^41^ that also used the WI-NRS score to characterize pruritus severity found that only 16% of patients on hemodialysis were classified as moderate-to-severe CKD-aP. This discrepancy may be explained by the fact that this study was conducted in an Austrian patient population, and according to the study by Rayner et al,^4^ Canada has a higher prevalence of patients on hemodialysis moderately to extremely bothered by itch (42%) compared with European countries (26% in Germany, 36% in Spain).

Within patients with moderate-to-severe CKD-aP, 29% classified themselves as SADS group A, 47% group B and 24% group C (most significant impact of CKD-aP on QOL). In the study by Engler et al,^41^ 15% of those with moderate-to-severe CKD-aP classified as SADS group A, 59% SADS group B, and 26% SADS group C. The differences in prevalence of SADS classifications between our study and theirs may relate to a lower threshold (WI-NRS score ≥ 4) to define moderate-to-severe pruritus. It is also important to note that in the study of Austrian patients,^41^ as WI-NRS score increased there was a greater prevalence of patients in SADS group C compared with our study. This suggests that the interpretation of the SADS scale and the impact of disease on QOL may differ between nations. This has not been explored in previous literature. It could be hypothesized that this occurs due to a greater clinical attention on pruritus symptom screening in European countries compared with Canada. In the study by Rayner et al,^4^ no medical directors in Canada refer more than half of their patients with pruritus to a dermatologist whereas in Germany, Spain and Sweden 6%, 6%, and 13% of medical directors respectively refer more than half of their patients with pruritus to a dermatologist. Ultimately, in both our study and Engler et al,^41^ around a quarter of patients with moderate-to-severe CKD-aP were severely debilitated by it. These results highlight the significant impacts of CKD-aP on QOL and therefore the importance of systematic symptom screening as recommended by the KDIGO.^17^ This will enable the identification of patients who may benefit from supportive care interventions focused on improving QOL.

Antihistamines were more commonly used amongst those with moderate-to-severe CKD-aP. Likewise, in the study by Rayner et al,^4^ antihistamines were the most common first line treatment amongst the majority of hemodialysis programs, as well as were most often used for patients who did not see a skin specialist. In our study, of those with moderate-to-severe CKD-aP, almost half did not use any form of treatment despite a quarter of them indicating pruritus interferes with their QOL. In addition, few used cannabinoids or were prescribed antihistamines. Within the group significantly affected by itch (SADS group C), 11% did not use any therapies, no patients were prescribed antihistamines or difellikafalen or used cannabinoids. A large number of patients in this study with CKD-aP used OTC antihistamines, despite the absence of substantial research showing its success in reducing pruritus. It thus is evident that there exists gaps and opportunities for care regarding the treatment of CKD-aP. Ultimately, a greater clinical focus must be placed on CKD-aP to effectively identify and treat symptoms. This study highlights the feasibility of using the WI-NRS and SADS scales to quantify the severity and prevalence of CKD-aP in a regional hemodialysis program, as well as identify patients in need of supportive therapies.

Body mass index was found to be weakly associated with WI-NRS score, and was associated with moderate-to-severe CKD-aP. The association between CKD-aP and BMI has been investigated in previous literature, however the majority of studies reported no significance.^5,7,12,41,42^ Patients with moderate-to-severe CKD-aP had a higher PTH and phosphate, and phosphate was associated with moderate-to-severe CKD-aP. This is supported by previous literature that also found significant associations between pruritus and elevated PTH and phosphate.^5,34,37,43^ However, other studies have also noted no associations between such variables.^41,44,45^ While ALP was correlated with moderate-to-severe CKD-aP in our study, previous literature has not reported this association.^46,47^ Finally, being a current smoker was associated with moderate-to-severe CKD-aP. However, there is not a great consensus amongst the literature regarding this association with several studies not investigating it,^7,36,42,47^ others also reporting an association,^12,48^ and another that reported no association.^49^

A limitation of this study is that there were missing data points as 5 participants who completed the WI-NRS did not complete the SADS, not all participants self-reported the use of OTC CKD-aP medications and there were some absent demographic and laboratory values in a few patient medical records. Another limitation is that we could not assess whether patients who classified as moderate-to-severe pruritus had CKD-aP or pruritus due to other reasons resulting in a possible over-estimation of the prevalence of CKD-aP. In addition, we could not identify treatment adherence, confirm the use of OTC medications reported by patients, nor could we be confident that the prescribed medication was for the indication of CKD-aP. Moreover, our dialysis electronic medical record system does not consistently capture prescribed topicals nor conditions commonly associated with itch. We thus could not report on either of these measures. Finally, this study did not assess physicians’ awareness of CKD-aP and whether routine symptom screening is performed. It is thus difficult to compare our results to studies conducted in other regions where clinical attention on CKD-aP may greatly differ.^5^

## Conclusion

This study assessed the prevalence and severity of CKD-aP in a regional hemodialysis program using validated tools. The prevalence of CKP-aP aligned with previously reported rates. The analysis revealed gaps in care as many patients with moderate-to-severe CKD-aP do not receive treatment. Due to the significant effects of CKD-aP on QOL, the KDIGO recommendations should be followed by introducing systematic screening for CKD-aP to develop care pathways designed to improve symptom management and QOL.

## Supplemental Material

Questionnaire Given to Study Participants Worst Itching Itensity Numerical Scale (WI-NRS) and Self-Assessed Disease Severity (SADS) scale given to study participants along with questions pertaining to self-reported use of over the counter medications.
