# Supplementary material for: Prevalence and Severity of Pruritus in Patients on Hemodialysis: A Cross-Sectional Study
Source: Can J Kidney Health Dis. 2025 Sep 28;12:20543581251380541. doi: 10.1177/20543581251380541 (PMC12477386; doi:10.1177/20543581251380541)
Supplement: Questionnaire Given to Study Participants [file sj-pdf-1-cjk-10.1177_20543581251380541.pdf]

NAME: ___________________________________

Worst-itch Numerical Rating Scale (WI-NRS)[1,2]

- **WI-NRS** is a validated **11-point scale to assess itch intensity** ranging from 0 - 10 where **0 represents 'no itching' and 10 'worst itch imaginable'**

Please rate the WORST ITCH you felt in the previous 24 hours

☐	☐	☐	☐	☐	☐	☐	☐	☐	☐	☐
**0**	**1**	**2**	**3**	**4**	**5**	**6**	**7**	**8**	**9**	**10**
No itching ☺										Worst itch imaginable ☹

Self-assessed Disease Severity (SADS) assesses the impact of itch on quality of life

Which of these patients are you most like? (Check profile)

○ **Patient A:**
- I do not generally have scratch marks on my skin
- I do not generally have a problem sleeping because of itching
- My itching does not generally make me feel agitated or sad

○ **Patient B:**
- I sometimes have scratch marks on my skin
- I sometimes have problems sleeping because of itching
- My itching can sometimes make me feel agitated or sad

○ **Patient C:**
- I often have scratch marks on my skin that may or may not bleed or get infected
- I often have a problem sleeping because of itching
- My itching often makes me feel agitated or sad

I use 'over the counter' treatments to help with itching? YES ☐ NO ☐

If **YES**, please list what you use: ___________________________________

____
